# Trigger wrist caused by avascular necrosis of the capitate: a case report

**DOI:** 10.1186/s12891-018-2010-1

**Published:** 2018-03-27

**Authors:** Yuichiro Matsui, Daisuke Kawamura, Hiroaki Kida, Kanako C. Hatanaka, Norimasa Iwasaki

**Affiliations:** 1 Department of Orthopaedic Surgery, Faculty of Medicine and Graduate School of Medicine, Hokkaido University, Kita-15 Nishi-7, Kita-ku, Sapporo, 060-8638 Japan; 2 Department of Surgical Pathology, Hokkaido University Hospital, Kita-14 Nishi-5, Kita-ku, Sapporo, 060-8648 Japan

**Keywords:** Trigger wrist, Avascular necrosis of the capitate, Capitolunate instability pattern

## Abstract

**Background:**

Trigger wrist is a rare condition first described by Marti in 1960, and various causes have been reported. The condition mostly occurs with finger flexion and extension, and rarely with flexion and extension of the wrist itself. Avascular necrosis of the capitate is also a rare condition, first described by Jönsson in 1942. While some reports of this condition have been published, little is known about its etiology. Therefore, no established treatment exists. We report a case of trigger wrist caused by avascular necrosis of the capitate.

**Case presentation:**

A 16-year-old right-handed male who was a high school handball player was referred to our department from a nearby hospital 5 months after the onset of pain in the dorsal aspect of the right wrist, with an unknown cause. At the previous hospital, imaging findings led to a diagnosis of avascular necrosis of the capitate, and conservative treatment with a wrist brace did not improve the pain. At the initial visit to our department, the patient was noted to have a painful trigger wrist that was brought on by wrist flexion and extension. Preoperative imaging findings led to a diagnosis of trigger wrist caused by capitolunate instability secondary to avascular necrosis of the capitate. We performed a partial excision of the proximal capitate with tendon ball interposition. Two years after surgery, the patient’s clinical outcome was favorable, with no recurrence of wrist pain or triggering.

**Conclusions:**

Both trigger wrist and avascular necrosis of the capitate are rare disorders. When a patient presents with painful triggering at the wrist, surgeons must bear in mind that avascular necrosis of the capitate may result in this phenomenon. We recommend partial excision of the proximal capitate with tendon ball interposition for the treatment of this lesion.

## Background

Trigger wrist is a rare condition that was first described by Marti in 1960 [1]. Since the first description, a variety of potential pathomechanisms have been suggested [2–11]. The condition mostly occurs with finger flexion and extension, and rarely with wrist motion. In published cases of trigger wrist caused by wrist flexion and extension, typical causes include muscular abnormality in the carpal tunnel region and a tumor or abnormal mass of the flexor tendon [2–5]. Avascular necrosis of the capitate is a rare condition, first described by Jönsson in 1942 [12]. While some reports of this disorder have been published [13–20], little is known about its etiology. Therefore, the optimal treatment for avascular necrosis of the capitate remains unknown. We report a case of trigger wrist caused by avascular necrosis of the capitate. To the best of our knowledge, this is the first report on the pathogenesis of trigger wrist caused by this condition in the English literature.

## Case presentation

**Fig. 1:**
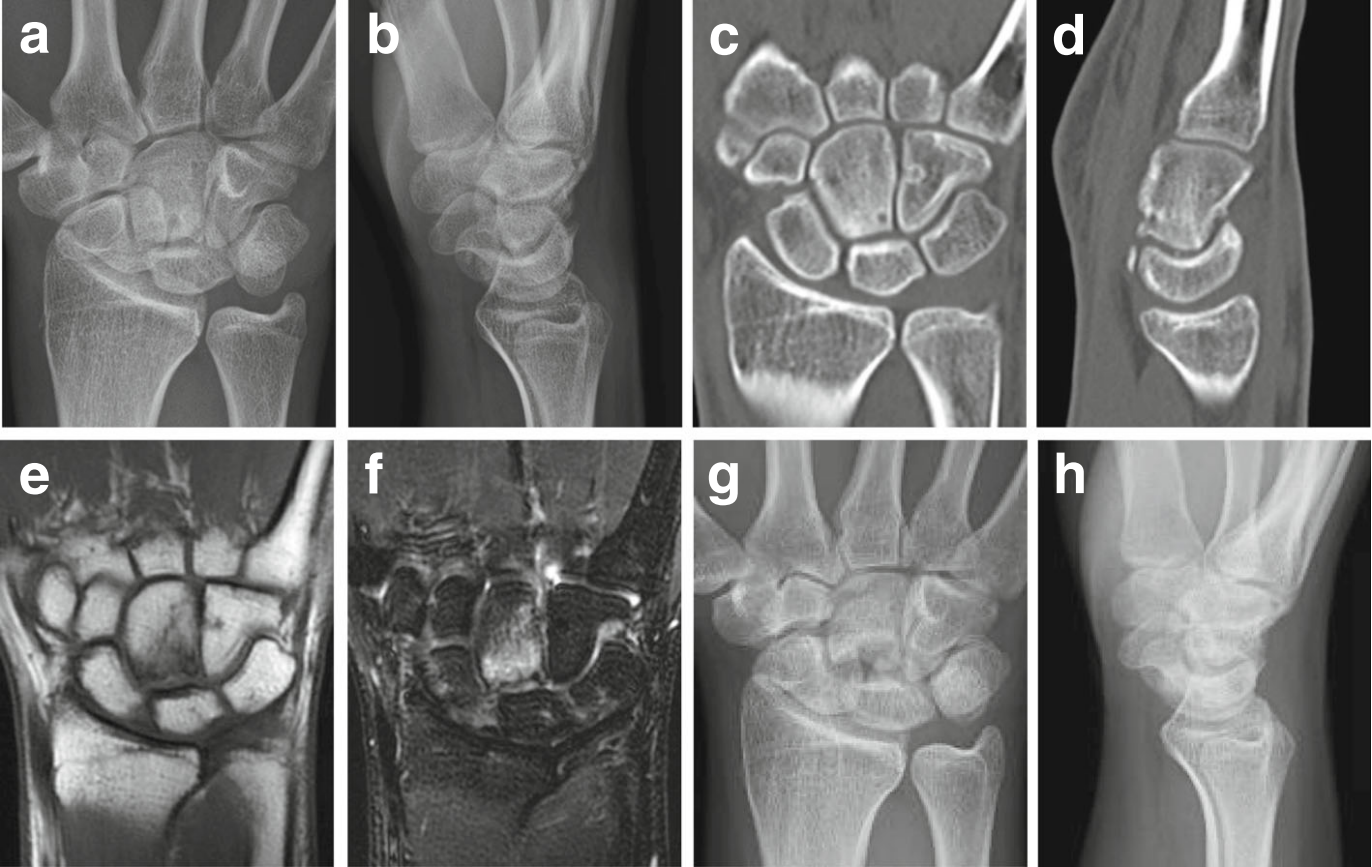
AP radiograph, CT, and MRI findings. **a**, **b** The preoperative radiographs showed collapse of the proximal portion of the capitate along with osteonecrosis. **c**, **d** The preoperative CT scans showed collapse of the proximal portion of the capitate along with osteonecrosis, a bone cyst in the proximal capitate, and a free body in the palmar proximal portion of the capitate. **e** A preoperative coronal T1-weighted MRI image showed low signal intensity at the proximal capitate. **f** A preoperative coronal STIR MRI image showed high signal intensity at the proximal capitate. **g**, **h** At the 2-year follow-up after surgery, the radiographs showed no further collapse of the capitate or progression of carpal instability

**Fig. 2:**
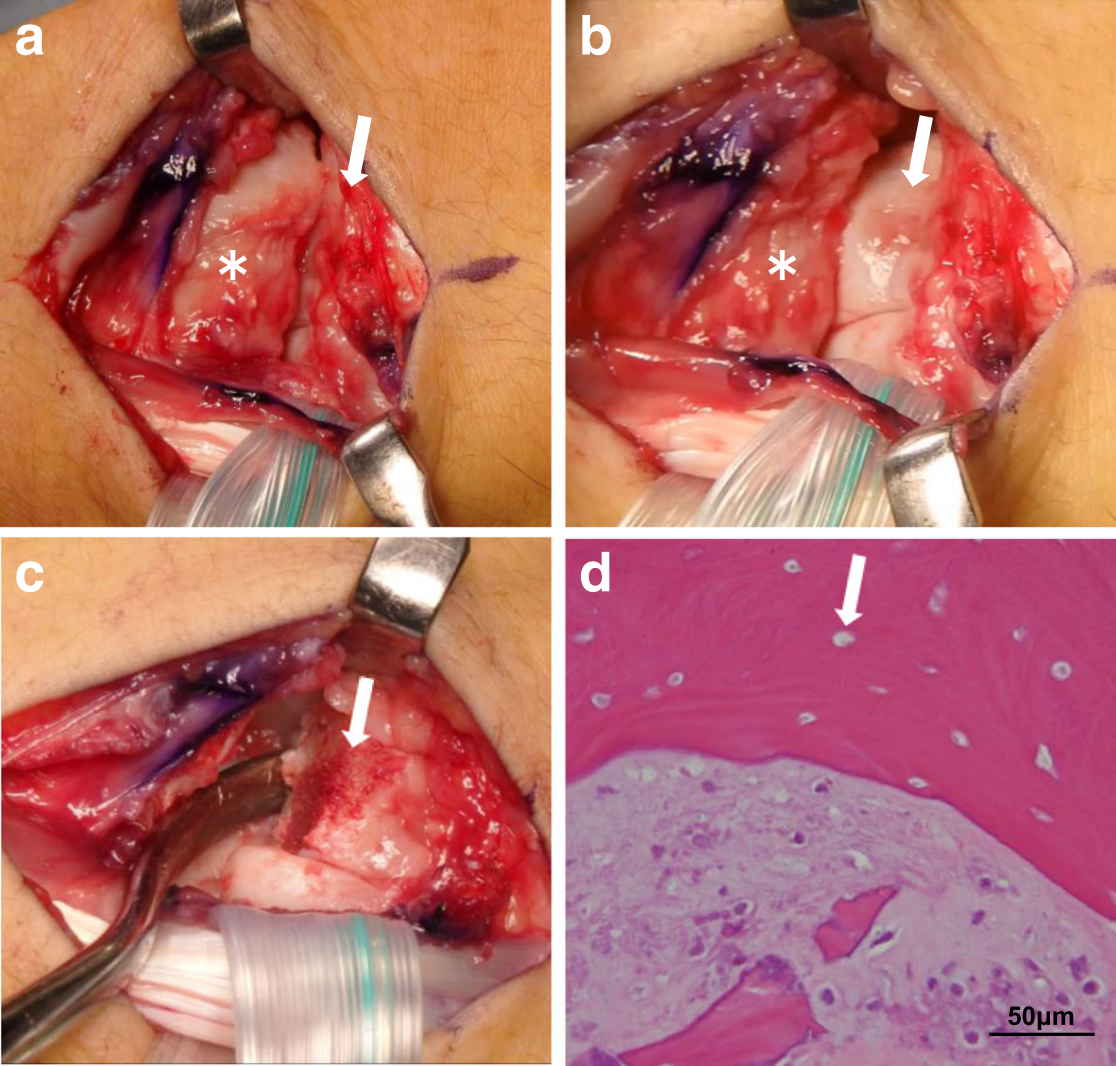
Intraoperative photographs and microscopic findings of the excised surgical specimen. **a** Intraoperative photograph of the capitate (arrow) and the lunate (asterisk) during passive wrist extension. **b** Intraoperative photograph showed the triggering phenomenon between the proximal capitate (arrow) and the lunate (asterisk) during passive wrist flexion. **c** Intraoperative photograph after partial resection of the capitate (arrow). **d** Hematoxylin and eosin staining of the excised specimen (magnification 40×) showed lack of osteocyte nuclei in the bone lacunae (arrow)

A 16-year-old right-handed male who was a high school handball player was referred to our department by a nearby hospital 5 months after the onset of pain of unknown etiology in the dorsal aspect of his right wrist. At the outside hospital, imaging findings led to a diagnosis of avascular necrosis of the capitate, but wrist immobilization using a brace did not improve the pain. At the initial visit to our department, the patient was noted to have mild swelling of the dorsal wrist, tenderness of the proximal capitate, and painful trigger wrist occurring with flexion and extension of the right wrist, with a range of motion in his wrist limited to 30°/60° of flexion/extension. The visual analog scale (VAS) score for pain was 71, with a grip strength of 70% relative to the unaffected side. Plain X-rays showed collapse of the proximal capitate and evidence of osteosclerosis, as well as palmar flexion of the lunate (Fig. 1a and b). The carpal height ratio and the radial lunate angle were 0.46 (unaffected side, 0.49) and 27° (unaffected side, 11°), respectively. The capitolunate angle was 32° (unaffected side, 24°). Computed tomography (CT) scans revealed a bone cyst of the proximal capitate and a free body from the palmar proximal capitate (Fig. 1c and d). On magnetic resonance imaging (MRI), the proximal capitate had a low signal intensity on T1-weighted images (Fig. 2e), and a high signal intensity on short TI inversion recovery (STIR) images (Fig. 2f). Fluoroscopic examination showed that the proximal articular surface of the capitate interfered with lunate motion during wrist flexion and extension. These findings led to a diagnosis of trigger wrist caused by capitolunate instability secondary to avascular necrosis of the capitate, for which surgery was performed. A longitudinal incision was made over the dorsal aspect of the right wrist, and the joint capsule was longitudinally incised over the capitate and lunate for exposure of the joint, revealing proliferation of inflammatory synovium in the joint. Morphological changes were noted on the proximal articular surface of the capitate, including flattening and eburnation. Intraoperative inspection revealed that limited compatibility between the capitate and lunate caused the triggering phenomenon during passive wrist flexion and extension (Fig. 2a and b). The proximal portion of the capitate was resected piece by piece using a bone chisel, while observing for any triggering during wrist flexion and extension. A palmaris longus muscle tendon ball was used to fill the cavity of the excised proximal capitate. The scaphoid and lunate were temporarily fixed using a Kirschner wire. A long-arm splint was applied for the first two weeks postoperatively, after which it was changed to a short-arm splint for an additional two weeks. Wrist rehabilitation was begun when the splint and the Kirschner wire used for fixation were removed at four weeks after surgery. Resumption of sports activities was permitted at 3 months postoperatively. Histopathological examination revealed a lack of osteocyte nuclei in the bone lacunae and incomplete ossification of the necrotic bone. These findings were consistent with avascular necrosis of the capitate (Fig. 2d). Two years after surgery, the patient’s clinical outcome was favorable, with no recurrence of wrist pain or triggering despite resumption of sports. He had an improved range of motion (75°/85° on flexion/extension) and increased grip strength (113% relative to the unaffected side). Plain X-rays showed no further collapse of the capitate, with a carpal height ratio of 0.49, a radial lunate angle of 10°, and a capitolunate angle of 18° (Fig. 1f and g).

## Discussion

The trigger wrist phenomenon is rare, though there have been several reports of it since its first description by Marti [1]. The term “trigger wrist” is defined as a painful click or catching sensation around the wrist joint during finger or wrist motion. However, the triggering is mostly induced by finger motion rather than motion of the wrist itself. Lemon and Engber introduced a distinction between triggering of the wrist specifically induced by finger motion and that induced by wrist motion [7]. Regarding the condition caused by finger motion, the reported causes include a muscular abnormality in the carpal tunnel region and a tumor or mass of the flexor tendon [1–5, 8, 10, 11]. On the other hand, the pathology caused by wrist motion includes extracapsular factors such as a nodule in the extensor carpi radialis longus tendon [7] and intra-articular factors. Intra-articular factors that can cause triggering are uncommon, but include scapholunate instability [6, 21], nondissociative carpal instability as seen with a capitolunate instability pattern [22, 23], and cartilaginous loose bodies within the radiocarpal joint [9]. Our intraoperative findings demonstrated the triggering phenomenon between the proximal capitate, including necrotic bone, and the lunate. To our knowledge, there have been no reports of trigger wrist caused by capitolunate instability secondary to avascular necrosis of the capitate.

Avascular necrosis of the capitate is a rare condition that was first reported by Jönsson et al. in 1942 [12], and little is known about its etiology. The intraosseous blood supply of the capitate has three patterns according to Grend et al. [24]. They reported that the blood supply to the proximal pole of the capitate depends on distal-to-proximal flow across the waist of the capitate. Milliez et al. proposed the following radiographic classification system for this condition based on the location of involvement in the capitate: type 1, the most common type, with necrosis involving the proximal pole; type 2, involving the distal body; and type 3, involving the entire capitate [15]. According to this system, the present case is classified as type 1. Regarding the etiology of avascular necrosis of the capitate, associations with impaired intraosseous blood flow, trauma, and steroid use have been suggested, but the details remain unclear. Murakami et al. suggested the possibility of osteonecrosis in gymnasts being caused by microfractures secondary to increased pressure in the wrist from repetitive wrist motion [14]. In our case, there was no history of specific trauma. However, the patient was an elementary and junior high school baseball player for six years and a high school handball player for six months. Therefore, repetitive wrist flexion and extension during the throwing motion could have placed stress on the wrist, leading to vascular insufficiency in the capitate.

To date, several surgical procedures have been recommended for the treatment of avascular necrosis of the capitate, including drilling, curettage of the partial excision of the capitate with tendon ball interposition, vascularized bone grafting, and intercarpal arthrodesis [13, 17, 18]. Drilling and vascularized bone grafting are indicated for avascular necrosis of the capitate that has not collapsed, or osteoarthritis of the midcarpal joint. Intercarpal arthrodesis is the most common surgical procedure for cases with collapse or osteoarthritis of the midcarpal joint. Although this procedure provides good pain relief for patients, it decreases the range of motion of the wrist [19]. For patients with no osteoarthritis of the midcarpal joint, partial excision of the proximal capitate with tendon ball interposition was considered to be the most effective treatment to preserve the range of wrist motion and prevent further triggering.

At 2 years after surgery, the patient has had no recurrence of wrist pain or triggering despite resumption of sports, with imaging showing no progression of capitolunate instability. Clinicians should be aware that avascular necrosis of the capitate typically occurs in young individuals involved in sports such as gymnastics, baseball, and handball. This lesion might result in capitolunate instability and associated onset of trigger wrist.

## Conclusions

We presented a case of trigger wrist caused by avascular necrosis of the capitate. Both trigger wrist and avascular necrosis of the capitate are rare disorders. When a patient presents with painful triggering at the wrist, surgeons must bear in mind that avascular necrosis of the capitate may result in this phenomenon. We recommend partial excision of the proximal capitate with tendon ball interposition for the treatment of this lesion.

## Abbreviations

CT: Computed tomography
MRI: Magnetic resonance imaging
STIR: Short TI inversion recovery
VAS: Visual analog scale
